# Efficacy and Safety of the Traditional Japanese Medicine Keigairengyoto in the Treatment of Acne Vulgaris

**DOI:** 10.1155/2018/4127303

**Published:** 2018-07-02

**Authors:** Kotaro Ito, Saori Masaki, Manabu Hamada, Tetsuo Tokunaga, Hisashi Kokuba, Kenji Tashiro, Ichiro Yano, Shinichiro Yasumoto, Shinichi Imafuku

**Affiliations:** ^1^Department of Dermatology, Fukuoka University School of Medicine, Fukuoka, Japan; ^2^Ito Dermatology Clinic, Oita, Japan; ^3^Hamada Dermatology Clinic, Fukuoka, Japan; ^4^Tokunaga Dermatology Clinic, Fukuoka, Japan; ^5^Sakurazaka Dermatology Clinic, Fukuoka, Japan; ^6^Tashiro Dermatology Clinic, Fukuoka, Japan; ^7^Yano Dermatology and Urinary Clinic, Fukuoka, Japan; ^8^Yasumoto Dermatology Clinic, Fukuoka, Japan

## Abstract

Several traditional Japanese medicines including Keigairengyoto (KRT) are used to treat acne vulgaris, but there is no robust evidence of their effectiveness. In this study, we examined the effectiveness and safety of KRT in treating acne vulgaris. An open-label, randomized, parallel control group comparison was conducted with a conventional treatment group (adapalene and topical antibiotics; control group) and a KRT group (control treatment plus KRT). The test drugs were administered for 12 weeks to patients (15 to 64 years, outpatient) with inflammatory acne on their face, and the amount of acne at 2, 4, 8, and 12 weeks was measured. Sixty-four patients were enrolled; 29 patients in each group were included in the analysis. Twenty-eight patients in the control group and 24 patients in the KRT group were included in the efficacy analysis. The number of inflammatory skin rashes at 4 and 8 weeks in the KRT group was significantly decreased compared with the control group. There was no significant difference between the two groups in noninflammatory eruptions and general rashes. There were no serious adverse events in both groups. KRT may be a useful agent in patients with inflammatory acne in combination with conventional treatments. This trial is registered with UMIN 000014831.

## 1. Introduction

Acne vulgaris is a chronic skin disorder in which a complex combination of abnormal lipid metabolism, abnormal keratosis, and proliferation of bacteria is involved in the pilosebaceous gland system. Acne starts with comedones (noninflammatory rash) with sebaceous secretion port obstruction resulting from abnormal proliferation and activation of keratinocytes and swelling of hair follicles. The disease progresses to pimples or pustules (inflammatory skin rashes) where acne bacteria and oxidized metabolites in the skin induce inflammation, accompanied by migration and infiltration of inflammatory cells. Exacerbation of inflammatory rash causes intractable conditions such as cysts and nodules, and the resulting scarring reduces the patient's quality of life. Therefore, it is important to prevent acne from progressing into inflammation. To achieve this, a quicker treatment response is required to improve patient compliance.

The main conventional treatment for acne consists of topical application of retinoid, benzoyl peroxide, and antibacterial agents [1]. The retinoid selectively binds to the retinoic acid receptor and suppresses keratinocyte proliferation and activation. A representative retinoid agent, adapalene, is used as a key drug during all stages of acne vulgaris. Benzoyl peroxide has a bactericidal action against* Propionibacterium acnes* and an anti-inflammatory action, and it produces few resistant bacteria. Recently, combination therapy using these drugs or a prescription drug combination was recommended [1]. Various antibiotics are positively prescribed when bacterial infection is the leading factor. However, adverse events such as skin irritation and resistant bacteria require a new treatment strategy [2–7].

In Japan, the traditional medication Keigairengyoto (KRT) in the form of extracted granules for ethical use (product number TJ-50; Tsumura & Co., Tokyo, Japan) has been approved for medicinal use by the Japanese Ministry of Health and Welfare and is widely prescribed for patients with inflammatory diseases including acne vulgaris, empyema, and rhinitis. The base powder of KRT was obtained by spray-drying a hot water extract mixture of the following 17 crude drugs: Scutellariae radix, Phellodendri cortex, Coptidis rhizoma, Platycodi radix, Aurantii fructus immaturus, Schizonepetae spica, Bupleuri radix, Gardeniae fructus, Rehmanniae radix, Paeoniae radix, Cnidii rhizoma, Angelicae radix, Menthae herba, Angelicae dahuricae radix, Saposhnikoviae radix, Forsythiae fructus, and Glycyrrhizae radix. KRT includes large amounts of various types of medicinal ingredients such as alkaloids, flavonoids, and triterpenoids, which exhibit antimicrobial, anti-inflammatory, antilipogenesis, and antioxidant effects [8–14]. It was recently reported that KRT suppressed development of bacteria-induced dermatitis in an experimental model, through enhancing bacterial clearance in innate immune cells [15]. However, no published clinical study has shown the efficacy of KRT. Our aim was to evaluate the efficacy and safety of KRT in a comparative study between the conventional treatment using adapalene and local antibiotics and this treatment combined with KRT therapy.

## 2. Methods

This study was a multicenter, open-label, randomized parallel group comparison study conducted at eight dermatologic medical facilities in Japan (UMIN000014831). This study was approved by the Ethics Review Committee of Fukuoka University Hospital.

### 2.1. Patients

Patients (15 to 64 years, male or female unconscious) with inflammatory acne who visited one of the eight facilities between August 2014 and January 2016 and agreed to participate in this research were enrolled. For patients under 18 years of age, consent was obtained from their parent or guardian. Exclusion criteria were as follows: (1) severe complications such as liver disease, renal disease, heart disease, blood disease, or metabolic disease; (2) being pregnant, lactating, or planning to become pregnant during the study observation period; (3) taking concomitant medications and research medicines within 1 week before the start of the study; (4) participation in another trial within one month before the initiation of this study; (5) scheduling to undergo a chemical peel or laser therapy during the study observation period; (6) a history of allergies to traditional Japanese medicine; and/or (7) patients for whom, in the opinion of the study scientist or collaborating research doctor, it is not in their best interest to be enrolled into the study.

### 2.2. Study Design

Patients were randomized into two groups: a conventional treatment group (control group) and a KRT plus conventional treatment group (KRT group). Patients in the control group received treatment with one of the antimicrobial agents (clindamycin gel or nadifloxacin cream) in addition to 0.1% adapalene gel. Vitamins (vitamin A, B2, B6, C, and E) were allowed in combination with the research medications. Antimicrobial oral medicine, herbal medicine, and hormonal drugs were prohibited as concomitant medications, and physical treatments such as a chemical peel and laser therapy were also prohibited. Patients in the KRT group received the conventional treatment mentioned above and Tsumura Keigairengyoto extract granules (Tsumura & Co.) divided into 7.5 g oral doses to be taken 2 to 3 times per day, before or between meals. Patients were treated for 12 weeks.

### 2.3. Assessments of Efficacy and Safety

The amount of inflammatory and noninflammatory acne on the face was counted at baseline (study entry) and at weeks 2, 4, 8, and 12. The reduction in this number was calculated for inflammatory, noninflammatory, and total acne. Adverse events including local and systemic symptoms were collected throughout the study period. Laboratory testing was performed before treatment and at 12 weeks (or at the time of discontinuation), and abnormal fluctuation was judged. The severity of adverse events was judged to be either “serious” or “not serious”.

### 2.4. Statistical Analysis

Evaluation of the effectiveness was performed by group and compared using the Wilcoxon rank-sum test, with the change from premedication to posttreatment as an index. Significance was set to 5% on both sides. Wilcoxon's signed-rank test was also used for intragroup analysis of longitudinal change.

## 3. Results

### 3.1. Patient Background

The patient background information for both groups is shown in Table 1. A total of 64 patients were enrolled: 31 in the control group and 33 in the KRT group. There were no differences between groups for age, sex, duration of disease, and severity of acne before treatment. There were a total of 58 patients in the full analysis set (FAS) and in the safety evaluation set (SES) (29 patients in the control group and 29 patients in the KRT group) and 52 patients in the efficacy analysis group (28 patients in the control and 24 patients in KRT group).

### 3.2. Reduction in the Amount of Acne

**Figure 1:**
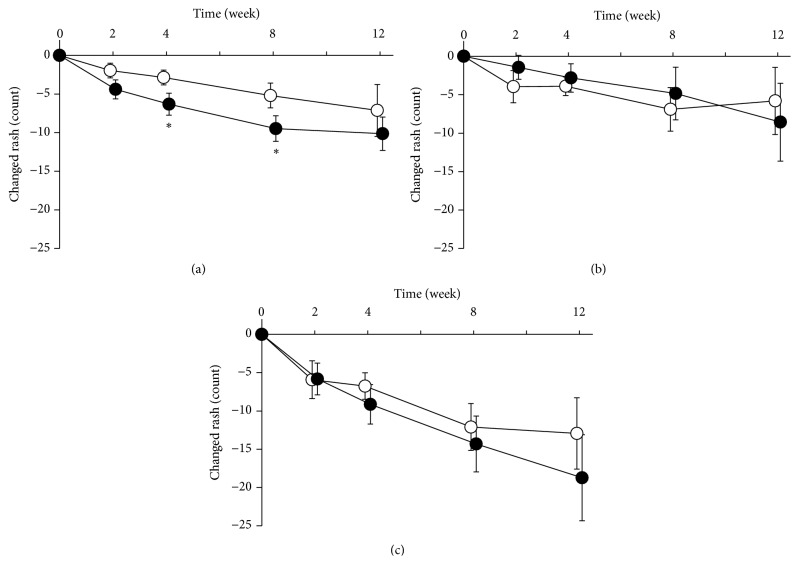
*Effect of Keigairengyoto on the number of acne rashes*. Patients were randomized into a conventional treatment group (control group, white circles) and a group with conventional treatment and Keigairengyoto (KRT group, filled circles), and they were treated for 12 weeks. The amount of inflammatory and noninflammatory acne on the face was counted at baseline (study entry) and at weeks 2, 4, 8, and 12. Time-dependent changes of inflammatory (a), noninflammatory (b), and total (c) acne are shown as a reduction in the number of the respective rashes. Data are presented as the mean ± standard error. The number of patients at the time of each control and KRT group evaluation is as follows: pretreatment: control 28 and KRT 24; week 2: 24 and 23; week 4: 20 and 22; week 8: 21 and 19; and week 12: 16 and 14. *∗*P<0.05, Wilcoxon rank-sum test.

In the control and KRT groups, the amount of acne decreased throughout the course of treatment (Figures 1(a), 1(b), and 1(c)). The amount of inflammatory acne in the KRT group declined significantly faster at 4 and 8 weeks compared with the control group. A significant difference was not observed in the amount of noninflammatory acne. There was no significant difference between the two groups in the total (inflammatory and noninflammatory) acne.

### 3.3. Representative Images

**Figure 2:**
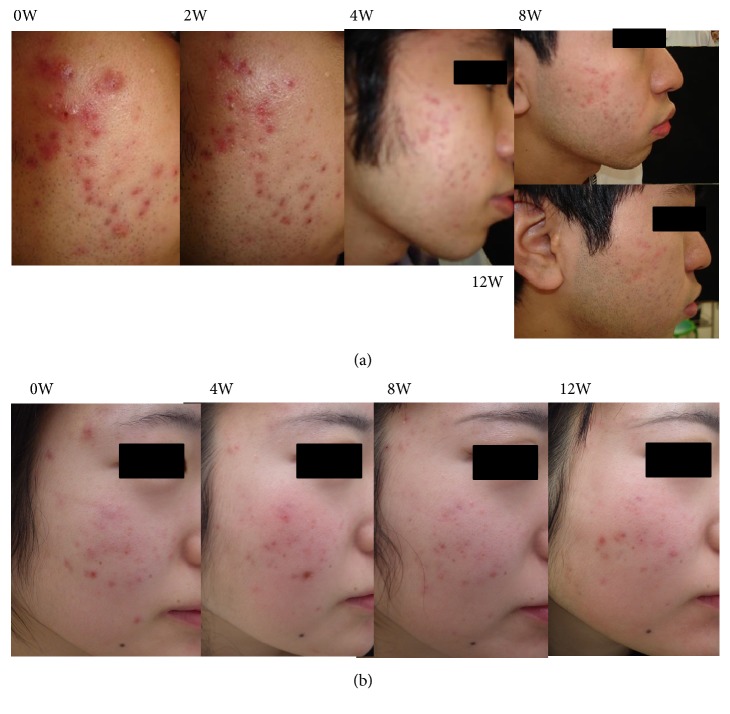
*Representative images of patients treated with Keigairengyoto*. Acne in two representative patients ((a) and (b)) treated with Keigairengyoto (KRT) at study entry and at weeks 2, 4, 8, and 12.

The treatment course of a representative patient with inflammatory acne in the KRT group is presented in Figures 2(a) and 2(b).

### 3.4. Stratified Analyses by Median Acne Duration

**Figure 3:**
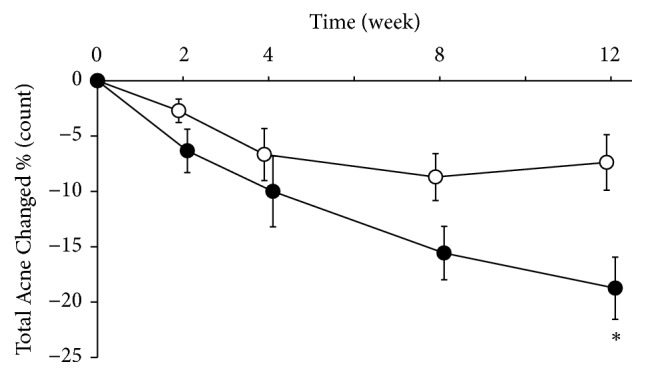
*Effect of Keigairengyoto in a stratified analysis focusing on patients with disease duration of 2.6 years or more*. Patients were randomized into a conventional treatment group (control group, white circle) and a group with conventional treatment and Keigairengyoto (KRT group, filled circle), and they were treated for 12 weeks. The median acne duration for all patients in this study was 2.6 years. The total sum of noninflammatory and inflammatory acne in patients with an acne duration of 2.6 years or more was examined. Although there was a difference in the number of patients among the groups, the KRT group showed significantly greater reduction in the amount of acne at week 12. For the change in inflammatory skin rash in patients with an acne duration of 2.6 years or more, the amount of inflammatory acne at 2, 4, and 8 weeks was lower in the KRT group compared with the control group, with a significant difference at 2 and 8 weeks. Data are shown as the mean ± standard error. The number of patients at each evaluation time point for the control and KRT groups is as follows: pretreatment: control 15 and KRT 9; week 2: 14 and 9; week 4: 9 and 9; week 8: 10 and 7; and week 12: 8 and 4. *∗*P<0.05, Wilcoxon rank-sum test.

The median acne duration in all patients in this study was 2.6 years. The total amount of noninflammatory and inflammatory acne in patients with a disease duration of 2.6 years or more was examined. Although there was a difference in the number of patients among the groups, the KRT group showed a significantly greater reduction in the amount of acne at week 12 (Figure 3).

### 3.5. Safety

Adverse events and adverse reactions from the 58 patients in the SES population were collected, and these are summarized in Table 2. In the control group, four adverse events in three patients were observed throughout the study period, three of which were local skin irritants, which seemed to be related to adapalene. In the KRT group, erosion of the lesion was seen in one of the 29 patients in the SES population. No event was serious.

## 4. Discussion

Although there have been clinical reports that traditional Japanese medicines are effective for treating acne [16], most of the research was not of high quality. In our multicenter, open-label, randomized controlled trial, KRT objectively showed the potential to reduce inflammatory acne at an early stage.

This study also suggests that KRT is not effective for noninflammatory acne and selectively suppresses inflammatory rashes. This selectivity suggests that KRT's point of action is different from that of adapalene or antibiotics. Because the amount of noninflammatory acne was not different at any time between the two groups, KRT may not affect keratinization abnormalities and sebaceous activity that subsequently cause comedones.

The significant point of this study is that adding KRT reduced the amount of inflammatory acne more than conventional therapy at 4 and 8 weeks. In treating acne, a rapid onset of the treatment effect is desirable to motivate patients to continue the therapy for this chronic condition.

Further stratification analyses were performed by duration of the acne. The total amount of noninflammatory and inflammatory acne in patients with a median disease duration (2.6 years or more) was examined. The KRT group showed a significantly greater reduction in the amount of acne at week 12 although there was a difference in the number of patients among the groups. For the change in inflammatory acne in patients with a disease duration of 2.6 years or more, the amount of acne at 2, 4, and 8 weeks was lower in the KRT group compared with the control group, with a significant difference at 2 and 8 weeks (data not shown). KRT may help patients more with longer history of inflammatory acne.

KRT is composed of the 17 crude drugs as described in the Introduction. Each component is identified by its external morphology and authenticated by marker compounds of plant specimens according to the methods of the Japanese Pharmacopoeia and quality standards defined at Tsumura Co. In the manufacturing process, KRT has been standardized with quantification of marker compounds including glycyrrhizin, berberine, and paeoniflorin and with specification levels for impurities such as heavy metal, pesticides, phytotoxins, and bacterial contamination under modern quality control. KRT has a large amount of various types of medicinal ingredients such as alkaloids (e.g., berberine), flavonoids (e.g., genistein, liquiritigenin, and baicalin), and triterpenoids (e.g., glycyrrhizin, saikosaponin) [8, 17–21]. Alkaloids and flavonoids are known to possess antimicrobial activity against a wide variety of microorganisms [8–10]. KRT exhibited the strongest effect in an antimicrobial assay using* P. acnes* among 10 kinds of kampo drugs that were evaluated [22]. According to a recent report, KRT drastically reduced the number of inoculated bacteria in a mouse cutaneous infection model using live* Staphylococcus aureus* [15]. It is unclear whether the antibacterial effect of KRT could be involved in clinical effects to ameliorate acne, but KRT may have the potential to affect an antibacterial spectrum in a manner that is different from the antibiotics used in the present study and/or to enhance the effects of the other drugs combined with KRT therapy.

A recent report showed that KRT's flavonoids, genistein and liquiritigenin, function as agonists for the nuclear estrogen receptor in macrophages, leading to enhancement of bacterial clearance by macrophages in both* in vivo* and* in vitro* assays [13]. KRT is reported to suppress bacteria-induced skin edema and enhanced bacteria phagocytosis by resident macrophages in a model of abscess-forming dermatitis induced by heat-killed* Staphylococcus aureus* [15]. Some flavonoids such as genistein and liquiritigenin were thought to contribute to the adjuvant effects of KRT. In acne patients' skin, resident macrophages play a host-defensive role to exclude* P. acnes* that is secreted via the pilosebaceous apparatus, dead cells, and debris generated by inflammation. If KRT can improve acne via upregulating macrophage functions, KRT is a unique drug that is different from the existing antiacne drugs.

Previous studies showed that flavonoids reduce development of contact or allergic dermatitis in experimental models [23, 24] and that tea flavonoids improve acne symptoms in humans [25]. Moreover, KRT is reported to show a radical scavenging effect in an assay using human peripheral white blood cells [26]. Blood pharmacokinetic and antioxidant studies focusing on KRT-related flavonoids [27] showed that flavonoids are important ingredients that contribute to the antiacne effect of KRT.

The limitations of this study are as follows: (1) there were a small number of patients; (2) the study was not double-blind, and thus conclusions based on these exploratory results should be made with caution. Larger studies that address these limitations are necessary in the future.

In summary, adding oral KRT onto conventional therapy significantly increased the reduction of inflammatory acne in the early stage of acne compared with conventional therapy alone. There was only one side effect in the KRT group during this study. KRT may be an option as a useful agent to treat inflammatory acne.

## Figures and Tables

**Table 1:** Background and number of acne patients.

	Control group	KRT group	Sum of the groups
Assigned number	31	33	64

FAS/SES number	29	29	58

PPS number			
Entry	28	24	52
2nd week	24	23	47
4th week	20	22	42
8th week	21	19	40
12th week	16	14	30

Age (year)	24.0±9.0	24.3±5.9	24.1±7.7

Gender M/F	12 / 16	9 / 15	21 / 31

Disease duration (year)	5.2±7.9	3.2±2.9	4.3±6.2

Severity poluration at entry (%)			
Mild	6 (21.4%)	3 (12.5%)	9 (17.3%)
Moderate	19 (67.9%)	18 (75.0%)	37 (71.2%)
Severe	2 (7.1%)	3 (12.5%)	5 (9.6%)
Very severe	1 (3.6%)	0	1 (1.9%)

Number of rash at entry			
Total rash	33.7±28.8	26.2±19.9	30.2±25.1
Inflammatory rash	14.6±22.3	12.5±9.9	13.7±17.6
Non-inflammatory rash	19.0±21.1	13.7±16.5	16.6±19.1

Patients were randomized into the conventional treatment group (control group) or the conventional treatment and Keigairengyoto group (KRT group), and they were treated for 12 weeks. Data on age, disease duration, and amount of acne at entry are shown as the mean ± standard deviation.

FAS, full analysis set; SES, safety evaluation set; PPS, per protocol set.

**Table 2:** Safety assessment.

	Control group				KRT group
	Patient #1	Patient #1	Patient #2	Patient #3	Patient #4
Adverse event	Dry skin	Erythema on right cheek	Cold	Xerotic eczema	Exacerbated rash

Severity	Not serious	Not serious	Not serious	Not serious	Not serious

Speculated drug	Adapalene	Adapalene	-	Adapalene	Keigairengyoto

Outcome	Improved	Improved	Recovered	Recovered	Recovered

Observation time^†^	1 week	1 week	3 weeks + 5 days	3 weeks + 1 day	4 days

Belonging to PPS	Yes	Yes	Yes	Yes	No

Patients were randomized into the conventional treatment group (control group) or the conventional treatment group with Keigairengyoto (KRT group), and they were treated for 12 weeks. Adverse events of local and systemic symptoms were collected throughout the study period.

^†^Observation time shows the duration from the start of treatment in the present study to finding an adverse event. PPS, per protocol set.

## Data Availability

The data used to support the findings of this study are available from the corresponding author upon request.
